# miRNA Expression Profiles of HPV-Infected Patients with Cervical Cancer in the Uyghur Population in China

**DOI:** 10.1371/journal.pone.0164701

**Published:** 2016-10-20

**Authors:** Dongmei Gao, Yuanyuan Zhang, Mingyue Zhu, Shuang Liu, Xinling Wang

**Affiliations:** Department of Gynecology, Cancer Hospital, Xinjiang Medical University, Urumqi, 830011, China; Fondazione IRCCS Istituto Nazionale dei Tumori, ITALY

## Abstract

The study aimed to investigate the state of human papillomavirus (HPV) infection in patients with cervical cancer in the Uyghur population in China and to identify miRNA as biomarker for cervical cancer and HPV infection. We also performed genotyping to determine the variation in the types of HPV. Using microRNA (miRNA) microarray technology, differential miRNA expression between HPV-infected cervical cancer and uninfected normal cervical tissues was determined; the microarray results were verified by quantitative reverse transcriptase polymerase chain reaction (qRT-PCR) using 20 samples of both the tissues. The infection rate of HPV in patients with cervical cancer was 96.7% (29 of 30), and the main subtype identified was HPV16 (29 of 29). HPV16 integration assay demonstrated that the majority of infectious cases were of the integrated form (26 of 29). Analysis of 140 miRNAs demonstrated greater than two-fold change in miRNA expression in HPV-infected cervical cancer tissue as compared to that in uninfected cervical tissue. The qRT-PCR analysis verified that the expression of miR-15a-5p, miR-17-5p, miR-20a-5p, miR-21-5p, miR-96, miR-106b-5p, and miR-3653 was higher, while the expression of miR-497-5p was lower in cancer tissues than in normal tissues. The results demonstrate significant changes in miRNA expression in cervical cancer tissues associated with HPV infection as compared to that in normal tissues. These molecular markers may be useful for an early diagnosis and prognosis of cervical cancer in specific human populations.

## Introduction

Cervical cancer is prevalent worldwide and is often fatal [1]. Although the factors leading to its occurrence are not fully understood, human papillomavirus (HPV) infection, as a causative agent of cervical cancer, has been well documented [2–4]. There are several subtypes of HPV, and those that cause cancers are generally referred as high-risk HPV (hrHPV). There are more than 20 common subtypes of hrHPV, with HPV16 being the most prevalent [5]. A previous study has shown a high degree of association between the carcinogenicity of hrHPV and its gene fragments *E6* and *E7* [6]. Protein translation from *E6* and *E7* is continuous throughout their integration into the host genome, which promotes cell proliferation and metastasis, leading to tumor progression [7, 8]. Disruption of *E2* is an important event leading to the development of cervical lesions [9]. A previous study has indicated that smaller the ratio of E2/E6, higher the number of malignant cervical lesions [10].

The Uyghur population is one of the largest minority populations in China, particularly in the Xinjiang Province. The infection rate of hrHPV in the Uyghur population is below the average rate in China [11]; however, the incidence and mortality rates of cervical cancer in this population are very high [12]. Uyghur population maintains a traditional lifestyle—women tend to marry earlier, have more children, and have poor health conditions. These reasons may result in a difference in the occurrence of cervical cancer in the Uyghur population in China. Therefore, addressing how cervical cancer develops in this population is of significant importance. Conventional treatments for cervical cancer lead to a poor prognosis due to a gradual increase in resistance to chemotherapy [13]. In recent years, the discovery of molecular biomarkers and the development of targeted therapy has improved the prognosis and quality of life of patients with cervical cancer [14]. Therefore, identifying molecular targets of cervical cancer in the Uyghur population has become a primary task, necessary for improving early diagnosis and prognosis of cervical cancer probably for the development of targeted therapy.

MicroRNAs (miRNAs) are a class of RNA molecules that play important roles in maintaining normal cellular function by regulating mRNA transcription. They are, generally, 18–25 nucleotides long, and can be found in various eukaryotes [15]. miRNAs play vital regulatory roles in cell growth, apoptosis, cell migration, and metastasis [16]. Moreover, they are also important in the development of cervical cancer. A previous miRNA microarray study demonstrated aberrant changes in miRNA expression in the cervical cancer tissues as compared to those in the normal tissues [17]. However, no study has investigated the changes in miRNA expression in the Uyghur population with HPV-infected cervical cancer as compared to that in the uninfected normal cervical tissue. Using miRNA microarrays, in this study, we evaluated the differences in miRNA expression in patients with HPV-infected cervical cancer and with uninfected normal cervical tissues within the Uyghur population. In addition, we validated our results using clinical samples, which allowed identification of miRNA with aberrant expression specific to the Uyghur population.

## Methods

### Patient and tissue samples

Cervical cancer tissues and uninfected normal cervical tissues were obtained from the Obstetrics and Gynecology Department of Xinjiang Medical University Cancer Hospital. All tissues were obtained between June 2012 and June 2013 as fresh specimens from surgical resection, and stored at -80°C until use. There were 30 cervical cancer tissues and 26 normal cervical tissues. Thirty cervical tissues were used to detect HPV infection—six cervical cancer tissues and six normal cervical tissues were used in miRNA microarray, and twenty cervical cancer tissues and twenty normal cervical tissues were used for validation of qPCR. The number of cases of cancer tissues from stage I to III is 5, 21, and 4, respectively. All patients belonged to the Uyghur population of Xinjiang Province, China. All cervical specimens were confirmed as squamous cell carcinoma through pathological examination. Prior to tissue sampling, patients had not received any chemotherapy treatment. Samples from the control group were determined to be normal by ThinPrep cytologic tests. This study was approved by the Ethics Committee of Xinjiang Medical University Cancer Hospital (K-201343), and an informed consent was obtained from all the patients.

### Detection of HPV infection

Total DNA was isolated from 30 cervical cancer tissues using the TIANamp Genomic DNA kit (Tiangen Biotech Co. Ltd, Beijing) according to the manufacturer’s instructions, and DNA concentrations were determined using NanoDrop 2000 spectrophotometer (Thermo Fisher scientific). PCR was performed on a gradient PCR instrument (Bio-Rad, USA), and the DNA polymerase used was Taq polymerase (Thermo Fisher Scientific). All PCR primers used are listed in Table 1. Detection of HPV infection was performed by applying MY09 and MY11 primers in the first round of PCR, and the PCR product was then diluted 1:100 before adding GP5+ and GP6+ primers to perform the second round of PCR [18]. For detection of HPV16 subtypes, GP-E6-3F and GP-E6-5R primers were used in the first round of PCR, and the PCR product was diluted 1:100 before adding HPV16Fz and HPV16Rz primers to perform the second round of PCR. The final product was electrophoresed on 1% agarose gel. For qPCR based on amplified fluorescence of multiple Taqman probes, the pCR-XL-TOPO-HPV16 plasmid (State Key Laboratory of Biological Resources and Genetic Engineering, School of Life Sciences and Technology, Xinjiang University) was used to construct a standard curve and was regarded as the positive control for HPV16 infection. Based on the amplification results of the standard HPV16 plasmid, HPV infection of each sample was determined to be either of an episomal or of an integrated form.

**Table 1:** Details of all primers used in the study.

Primer name	Sequence (5′ to 3′)	Number of bases	Length of PCR product (bp)
MY09	CGTCCMARRGGAWACTGATC	20	450
MY11	GCMCAGGGWCATAAYAATGG	20	
GP5+	TTTGTTACTGTGGTAGATACTAC	23	150
GP6+	GAAAAATAAACTGTAAATCATATTC	25	
GP-E6-3F	GGGCGKAACTGAAATCGGT	19	609
GP-E6-5R	CTGAGCTGTCATTTAATTGCTCA	23	
HPV16Fz	CACAGTTATGCACAGAGCTGC	21	457
HPV16Rz	CATATATTCATGCAATGTAGGTGTA	25	
HPV16 E2-F	GAAACACAGACGACTATCCA	20	
HPV16 E2-R	TCCGTCCTTTGTGTGAGCTGT	21	
HPV16 E2 TaqMan probe	HEX-CCAAGATCAGAGCCAGACAC-Eclipse	20	
HPV16 E6-F	GAATGTGTGTACTGCAAGCA	20	
HPV16 E6-R	GTTGTATTGCTGTTCTAATGTTGT	24	
HPV16 E6 TaqMan probe	FAM-CAGCATATGGATTCCCATCTC-Eclipse	23	
hsa-miR-106b-5p-F	UAAAGUGCUGACAGUGCAGAU	21	
hsa-miR-106b-5p-R	GCGAAGAGGTGACAGTGCAGAT	22	
hsa-miR-3188-F	AGAGGCUUUGUGCGGAUACGGGG	23	
hsa-miR-3188-R	GCGAGAGAGTCTTTGTGCGGATAC	24	
hsa-miR-3653-F	CUAAGAAGUUGACUGAAG	18	
hsa-miR-3653-R	GCGACGCTAAGAAGTTGACTGAAG	24	
hsa-miR-497-5p-F	CAGCAGCACACUGUGGUUUGU	21	
hsa-miR-497-5p-R	CGACAGCAGCACACTGTGGTT	21	
hsa-miR-590-5p-F	GAGCUUAUUCAUAAAAGUGCAG	22	
hsa-miR-590-5p-R	GCCGACCTTATTCATAAAAGTGCAG	25	

### miRNA microarray analysis

Six cervical cancer tissues infected with HPV16 along with six normal cervical tissues were randomly selected. Total RNA was isolated using the TRIzol Reagent (Invitrogen) according to the manufacturer’s instructions and total RNA was treated with DNase. RNA concentrations were determined by NanoDrop 2000 spectrophotometer (Thermo Fisher Scientific). RNA quality and integrity were examined using an Agilent 2100 Bioanalyzer (Agilent Technologies, USA). All RNA samples used for miRNA microarrays exhibited a RIN (RNA integrity number) of ≥ 6.0, and ratio of 28S/18S ≥ 1.7. Subsequently, reverse transcription (RT) was performed on these samples using 1.0 μg of total RNA to generate cDNA using the miScript II RT kit (QIAGEN, Germany).

Labeling and hybridization experiments were conducted by Urumqi OE Biotech Company, according to the protocol of Agilent human 8×60K miRNA microarray system. Microarrays were scanned using the Agilent Scan Control software, and all analyses were performed using the Agilent Feature Extraction software (version 10.7.1.1, Agilent Technologies). Raw data were normalized using the Quantile algorithm and Gene Spring Software (Agilent Technologies, USA).

### Quantitative real time polymerase chain reaction analysis (qRT-PCR)

Cervical cancer tissues infected with HPV16 and normal cervical tissues were randomly selected (n = 20/group), and total RNA was isolated using the TRIzol reagent (Invitrogen, USA), according to the manufacturer’s instructions. Total RNA was treated with DNase and RNA concentrations were determined using a NanoDrop 2000 spectrophotometer (Thermo Fisher scientific). Total RNA (1 μg) was converted into cDNA using the miScript II RT kit (QIAGEN, Germany). qPCR was performed using SYBR Select Master Mix reagent (Invitrogen) on an ABI Prism 7500 detection system (Invitrogen). The PCR conditions were as follows: denaturation at 95°C for 15 sec, followed by annealing and extension at 60°C for 1 min. The primers for miR-218-5p, miR-17-5p, miR-96, miR-15a-5p, miR-20a-5p, and miR-21-5p were obtained from Tiangen Biotech Co. Ltd (Beijing, China). The other primers are listed in Table 1. miRNA expression level was calculated using the 2^-ΔΔC^_t_ method, the internal control used was U6.

### Statistical analysis

Differential miRNA expression was based on the following three conditions: (1) miRNA was expressed in at least 75% of the samples; (2) the difference in the miRNA expression between the two groups was equal to or greater than two-fold change; and (3) the Student’s *t*-test between the two groups yielded a p-value of < 0.05. All microarray assays were expressed as log2 of microarray signals,and a multiple correction test was applied to microarray data.

The results of the qRT-PCR assay were analyzed using the SPSS 17.0 software (IBM, USA). All experiments were repeated thrice. Data were expressed as mean ± standard deviation. Statistical differences between the two groups were determined by Student’s *t*-test. Differential expression was considered statistical significant at a p-value < 0.05 between the two groups.

## Results

### Patient data

The samples used for miRNA microarrays were from six cervical cancer tissues infected with HPV16 and six uninfected normal cervical tissues. The average age of the patients in the cervical cancer group was 58 years 7 months (range: 53–64 years), and the average age of those in the control group was 40 years 7 months (range: 28–45 years). There was no significant difference in the age between these two groups (p > 0.05). For qRT-PCR experiments, 20 cervical cancer tissues infected with HPV16 and 20 uninfected normal cervical tissues were analyzed. The average age of the patients in the cervical cancer group was 47 years 7 months (range: 41–62 years), and the average age of those in the control group was 44 years 5 months (range: 25–69 years). There was no statistically significant difference in the age between these two groups (p > 0.05). All the samples were obtained from women belonging to the Uyghur population.

### High detection rate of HPV16 in cervical cancer tissues from the Uyghur population

**Fig 1:**
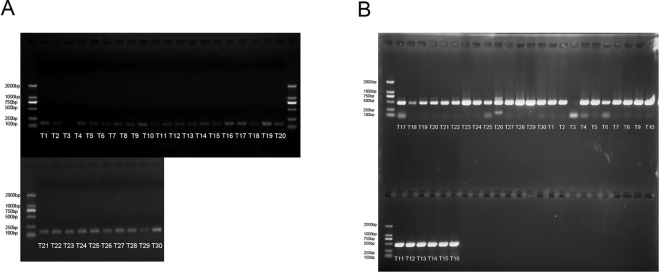
(A) Identification of cervical cancer tissues infected with HPV; (B) Identification of HPV genotype. The target bands are located at 150 bp and 457 bp.

We initially examined the infection state of HPV in the cervical cancer tissues of 30 patients. The DNA electrophoresis results showed that 29 of the 30 patients had HPV infection, with an infection rate of 96.67% (Fig 1A). Subsequent HPV genotyping revealed that 29 cases were caused by the HPV16 subtype. These results demonstrate the high HPV infection rate in patients with cervical cancer within the Uyghur population in Xinjiang Province (Fig 1B). Among the 29 cases of HPV16 infection, the expression of *E6* was higher than that of *E2* in 26 cases (Table 2), which suggests that these infections were of the integrated form.

**Table 2:** The status of HPV16 infection of 29 patients with cervical cancer.

case	expression of E2	expression of E6	Ratio(E2 /E6)	status
T1	196.5367652	640.0229518	0.307077683	integrated
T2	254075.384	332267.4344	0.764671339	integrated
T4	4143353.075	9174234.224	0.45162931	integrated
T5	229864.7201	971148.8077	0.236693613	integrated
T6	38685.87163	121544.2711	0.318286261	integrated
T7	12768525.01	21444957.29	0.595409207	integrated
T8	2592824.161	5290079.961	0.490129484	integrated
T9	27341.7162	580030.2212	0.047138434	integrated
T10	1016.361072	2259.709895	0.449775024	integrated
T11	325937.6378	1238904.809	0.263085296	integrated
T12	270.5257598	22545.03815	0.011999348	integrated
T13	464698.7518	328704.5304	1.413727858	dissociated
T14	2517.288164	388929.2755	0.006472355	integrated
T15	60.98979222	34909.30275	0.001747093	integrated
T16	2086187.558	16694902.81	0.124959551	integrated
T17	875.2390537	112046.0591	0.007811422	integrated
T18	1275.454153	5681.330491	0.2244992	integrated
T19	9012165.704	14284546.84	0.630903157	integrated
T20	668.2854947	2289.563661	0.291883343	integrated
T21	28904.79162	145312.2892	0.198914984	integrated
T22	97.48535055	7198.336394	0.013542761	integrated
T23	1330101.242	3827263.998	0.347533184	integrated
T24	6095.375778	37592284.47	0.000162144	integrated
T25	57.54174018	2937.976581	0.0195855	integrated
T26	136.4644152	21049.88812	0.006482905	integrated
T27	694.3840603	46472.76647	0.014941741	integrated
T28	1890.882282	19456.45244	0.097185357	integrated
T29	171146.3166	97057.28286	1.763353677	dissociated
T30	87743.32766	46407.93468	1.890696672	dissociated

### miRNA microarray analysis of HPV-infected cervical cancer tissues and uninfected normal cervical tissues

**Fig 2:**
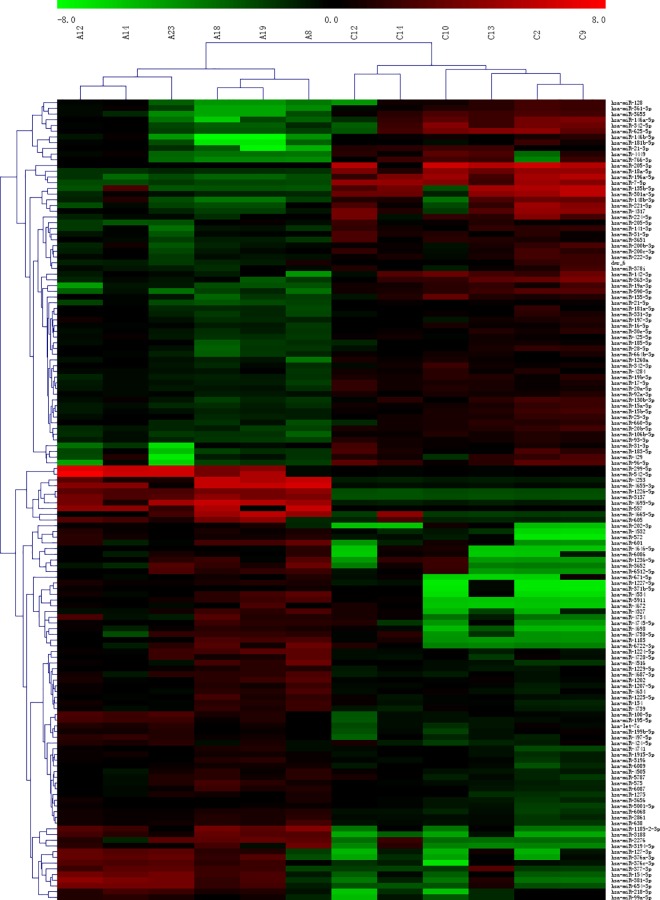
Cluster analysis of miRNA microarrays for cervical cancer and normal cervical tissues.

Of 2028 miRNAs, 140 demonstrated at least 2-fold change in expression in cancer tissues infected with HPV16 as compared to those in normal cervical tissues. Among these, the expression of 64 miRNAs was higher and 76 miRNAs was lower in cancer cells (Fig 2).

### Validation of differentially expressed genes by qRT-PCR analysis

**Fig 3:**
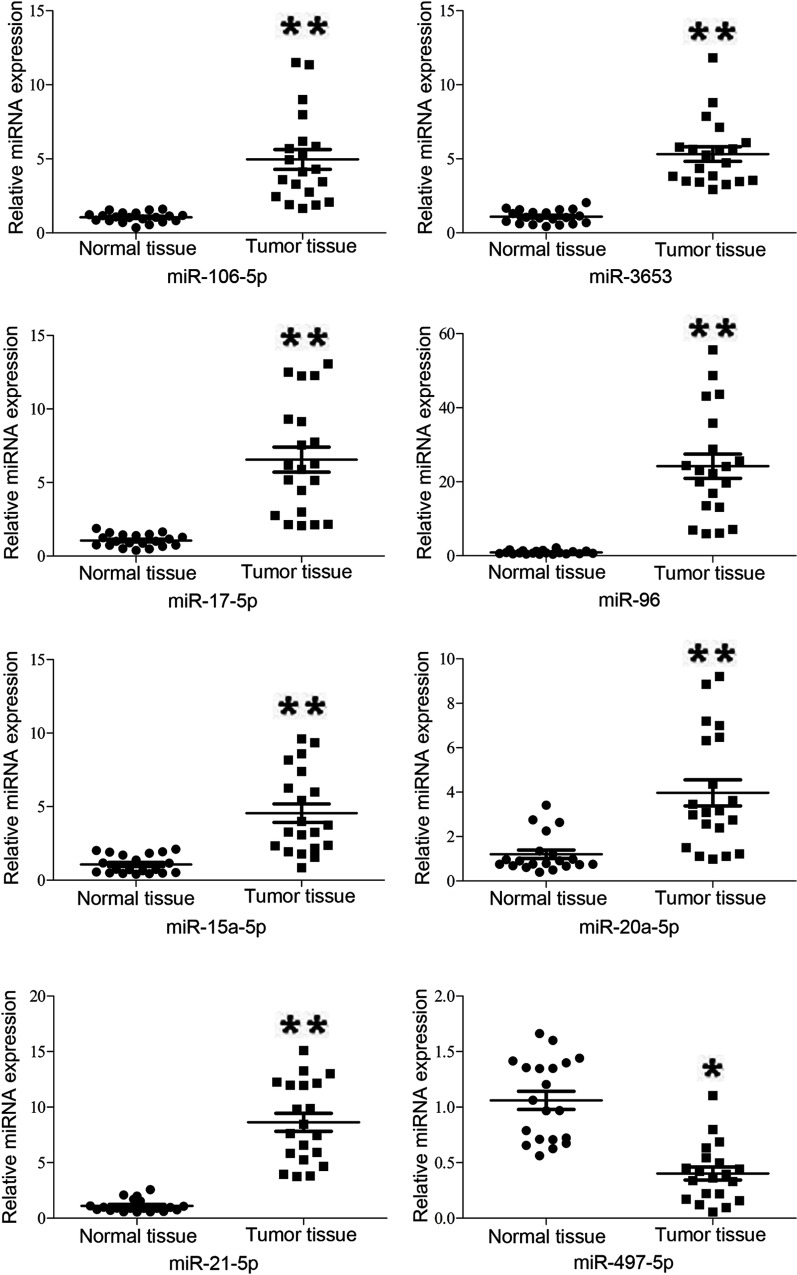
Relative quantification of differentially expressed miRNAs in 20 cervical cancer and 20 normal cervical tissues. qPCR analysis was performed in three separate experiments (*p < 0.05; **p < 0.01).

qRT-PCR analysis was performed on 11 randomly selected miRNAs that were differentially expressed based on the microarray evaluation of 20 HPV-infected cervical cancer and 20 uninfected normal cervical tissues. These miRNAs included miR-106b-5p, miR3653, miR-3188, miR-497-5p, miR-218-5p, miR-17-5p, miR-96, miR-15a-5p, miR-20a-5p, miR-21-5p, and miR-590-5p (Table 3). The qRT-PCR results demonstrated that miR-106b-5p (p = 0.000), miR-3653 (p = 0.000), miR-17-5p (p = 0.000), miR-96 (p = 0.000), miR-15a-5p (p = 0.000), miR-20a-5p (p = 0.000), and miR-21-5p (p = 0.000) were highly expressed in cancer tissues, while miR-497-5p (p = 0.016) was expressed at very low levels. However, the expression changes in miR-3188 (p = 0.975), miR-218-5p (p = 0.213), and miR-590-5p (p = 0.193) were not statistical significant between the two groups. Among the 11 miRNAs, the expression of 8 miRNAs matched the microarray results, thus yielding a consistency rate of 72.7% (Fig 3).

**Table 3:** Change in miRNA expression by miRNA microarray.

miRNA name	FC (abs)	p	Regulation
hsa-miR-106b-5p	5.273432	7.26E-05	up
hsa-miR-15a-5p	4.357547	2.65E-05	up
hsa-miR-17-5p	3.515523	9.27E-05	up
hsa-miR-20a-5p	3.171946	5.40E-05	up
hsa-miR-21-5p	4.823086	1.13E-04	up
hsa-miR-3653	30.34445	2.92E-04	up
hsa-miR-590-5p	16.09113	7.68E-05	up
hsa-miR-96-5p	30.82394	8.85E-04	up
hsa-miR-218-5p	12.1698	0.015844	down
hsa-miR-3188	74.07616	3.30E-05	down
hsa-miR-497-5p	3.595281	0.005715	down

## Discussion

Using miRNA microarray followed by qRT-PCR, we identified miRNAs related to the development of HPV-associated cervical cancer in women belonging to the Uyghur population. Through interaction with the 3′UTR region of mRNA, miRNA inhibits mRNA function at the transcriptional level. Here, we identified eight types of miRNAs, namely, the highly expressed miR-15a-5p, miR-17-5p, miR-20a-5p, miR-21-5p, miR-96, miR-106b-5p, and miR-3653 as well as the poorly expressed miR-497-5p. The aberrant expression of these miRNAs could have a role in the development of cervical cancer.

Some microarray studies have reported an abnormal expression of miRNA in cervical cancer. A total of 18 miRNAs were screened in the pre-invasive and invasive stages [19]. In another study, 9 miRNAs, including miR-218 and miR-21, were validated by qPCR between different stages of cervical neoplasm [20]. These studies indicated that miRNA played an important role in the progress of cervical cancer.

Among the identified miRNAs, the differential expression of miR-96 was the most pronounced (FC = 26.31), and its overexpression has been observed in several cancers [21, 22]. Furthermore, it has been reported that the expression of miR-96 is significantly increased during cervical cancer metastasis [23], suggesting its possible role in the metastatic process.

HPV DNA often integrates into the host genome after infection. A recent study demonstrated that persistent HPV infection and disease progression harbored the integrated form of HPV16 DNA rather than the episomal form [24]. *E6/E7* are responsible for cancer development. The expression of miRNAs, including those regulated by miR-15a-5p, could be upregulated via the interaction of E6 with c-MYC [25, 26]. Moreover, E6 can degrade *p53* [27, 28], thus upregulating the expression of miR-17-5p, miR-20a-5p, and miR-106b-5p [29]. E7 can mediate the degradation of *pRB* [30], which reduces the pRB-E2F1 complex and increases the expression of the transcription factor E2F1, resulting in an increase in miRNAs, including miR-106b-5p [31]. miR-21 expression can also be affected by E6/E7, as these viral proteins can upregulate the expression of the transcriptional activator of miR-21, p65 NF-κB [32, 33]. These findings imply that miR-15a-5p, miR-17-5p, miR-20a-5p, miR-21-5p, and miR-106b-5p are molecular targets of HPV *in vivo*.

In addition, miR-497 can inhibit the proliferation of cervical cancer cells by acting on cyclin E1 [34]. miR-497 is also closely associated with the prognosis of many types of cancers, such as kidney and pancreatic cancers [35, 36]. As it is a newly discovered miRNA, there have been very few reports on miR-3653, and further studies are needed to determine its role in the development of cervical cancer.

In this study, we were unable to examine miRNA expression in different stages and types of cervical cancer due to limited samples. Thus, further research will focus on overcoming this limitation through the involvement of multiple medical centers and large-scale studies.

In conclusion, we identified a series of miRNAs that can be used to detect HPV infection associated with cervical cancer in the Uyghur women of Xinjiang Province. These miRNAs provide new insight into the molecular basis of cervical cancers. They can therefore serve as biomarkers to improve early diagnosis, prognosis, and treatment of cervical cancers.

## Supporting Information

S1 Protocol Trial protocol. (DOCX)

## References

[1] Torre LA, Bray F, Siegel RL, Ferlay J, Lortet-Tieulent J, Jemal A. Global cancer statistics, 2012. CA: a cancer journal for clinicians. 2015;65(2):87–108. 10.3322/caac.21262 . 25651787

[2] Munoz N, Bosch FX, de Sanjose S, Herrero R, Castellsague X, Shah KV, et al Epidemiologic classification of human papillomavirus types associated with cervical cancer. The New England journal of medicine. 2003;348(6):518–27. 10.1056/NEJMoa021641 . 12571259

[3] Walboomers JM, Jacobs MV, Manos MM, Bosch FX, Kummer JA, Shah KV, et al Human papillomavirus is a necessary cause of invasive cervical cancer worldwide. The Journal of pathology. 1999;189(1):12–9. Epub 1999/08/19. 10.1002/(sici)1096-9896(199909)189:1<12::aid-path431>3.0.co;2-f . 10451482

[4] Bosch FX, Lorincz A, Munoz N, Meijer CJ, Shah KV. The causal relation between human papillomavirus and cervical cancer. Journal of clinical pathology. 2002;55(4):244–65. Epub 2002/03/29. ; PubMed Central PMCID: PMCPmc1769629. 11919208 10.1136/jcp.55.4.244 PMC1769629

[5] Crosbie EJ, Einstein MH, Franceschi S, Kitchener HC. Human papillomavirus and cervical cancer. Lancet. 2013;382(9895):889–99. 10.1016/S0140-6736(13)60022-7 . 23618600

[6] Obeidat B, Matalka I, Mohtaseb A, Jaradat S, Hayajneh W, Khasawneh R, et al Prevalence and distribution of high-risk human papillomavirus genotypes in cervical carcinoma, low-grade, and high-grade squamous intraepithelial lesions in Jordanian women. European journal of gynaecological oncology. 2013;34(3):257–60. . 23967558

[7] Moody CA, Laimins LA. Human papillomavirus oncoproteins: pathways to transformation. Nature reviews Cancer. 2010;10(8):550–60. 10.1038/nrc2886 . 20592731

[8] Narisawa-Saito M, Kiyono T. Basic mechanisms of high-risk human papillomavirus-induced carcinogenesis: roles of E6 and E7 proteins. Cancer science. 2007;98(10):1505–11. 10.1111/j.1349-7006.2007.00546.x . 17645777 PMC11158331

[9] Cheung JL, Lo KW, Cheung TH, Tang JW, Chan PK. Viral load, E2 gene disruption status, and lineage of human papillomavirus type 16 infection in cervical neoplasia. The Journal of infectious diseases. 2006;194(12):1706–12. 10.1086/509622 . 17109343

[10] Cricca M, Morselli-Labate AM, Venturoli S, Ambretti S, Gentilomi GA, Gallinella G, et al Viral DNA load, physical status and E2/E6 ratio as markers to grade HPV16 positive women for high-grade cervical lesions. Gynecologic oncology. 2007;106(3):549–57. 10.1016/j.ygyno.2007.05.004 . 17568661

[11] Li J, Li LK, Ma JF, Wei LH, Niyazi M, Li CQ, et al Knowledge and attitudes about human papillomavirus (HPV) and HPV vaccines among women living in metropolitan and rural regions of China. Vaccine. 2009;27(8):1210–5. Epub 2009/01/13. 10.1016/j.vaccine.2008.12.020 . 19135493

[12] Jiang S, Wang T, Tu S, Zhou J, Mairemu S, Xu X, et al Epidemiological investigation of cervical cancer in Xinjiang Cele County. Chin J Pract Gynecol Obstet. 2006;20:379–381.

[13] Liu S, Pan X, Yang Q, Wen L, Jiang Y, Zhao Y, et al MicroRNA-18a enhances the radiosensitivity of cervical cancer cells by promoting radiation-induced apoptosis. Oncology reports. 2015;33(6):2853–62. 10.3892/or.2015.3929 . 25963391

[14] de Freitas AC, Gomes Leitao Mda C, Coimbra EC. Prospects of molecularly-targeted therapies for cervical cancer treatment. Current drug targets. 2015;16(1):77–91. . 25479546 10.2174/1389450116666141205150942

[15] Bartel DP. MicroRNAs: target recognition and regulatory functions. Cell. 2009;136(2):215–33. 10.1016/j.cell.2009.01.002 19167326 PMC3794896

[16] Gardiner AS, Twiss JL, Perrone-Bizzozero NI. Competing Interactions of RNA-Binding Proteins, MicroRNAs, and Their Targets Control Neuronal Development and Function. Biomolecules. 2015;5(4):2903–18. 10.3390/biom5042903 . 26512708 PMC4693262

[17] Lee JW, Choi CH, Choi JJ, Park YA, Kim SJ, Hwang SY, et al Altered MicroRNA expression in cervical carcinomas. Clinical cancer research: an official journal of the American Association for Cancer Research. 2008;14(9):2535–42. 10.1158/1078-0432.CCR-07-1231 . 18451214

[18] Fuessel Haws AL, He Q, Rady PL, Zhang L, Grady J, Hughes TK, et al Nested PCR with the PGMY09/11 and GP5(+)/6(+) primer sets improves detection of HPV DNA in cervical samples. Journal of virological methods. 2004;122(1):87–93. 10.1016/j.jviromet.2004.08.007 . 15488625

[19] Zhu XL, Wen SY, Ai ZH, Wang J, Xu YL, Teng YC. Screening for characteristic microRNAs between pre-invasive and invasive stages of cervical cancer. Mol Med Rep. 2015;12(1):55–62. 10.3892/mmr.2015.3363 . 25695263 PMC4438941

[20] Zeng K, Zheng W, Mo X, Liu F, Li M, Liu Z, et al Dysregulated microRNAs involved in the progression of cervical neoplasm. Archives of gynecology and obstetrics. 2015;292(4):905–13. 10.1007/s00404-015-3702-5 . 25851497

[21] Xia H, Chen S, Chen K, Huang H, Ma H. MiR-96 promotes proliferation and chemo- or radioresistance by down-regulating RECK in esophageal cancer. Biomedicine & pharmacotherapy = Biomedecine & pharmacotherapie. 2014;68(8):951–8. 10.1016/j.biopha.2014.10.023 . 25465153

[22] Wu Z, Liu K, Wang Y, Xu Z, Meng J, Gu S. Upregulation of microRNA-96 and its oncogenic functions by targeting CDKN1A in bladder cancer. Cancer cell international. 2015;15:107 10.1186/s12935-015-0235-8 26582573 PMC4650312

[23] Ding H, Wu YL, Wang YX, Zhu FF. Characterization of the microRNA expression profile of cervical squamous cell carcinoma metastases. Asian Pacific journal of cancer prevention: APJCP. 2014;15(4):1675–9. . 24641388 10.7314/apjcp.2014.15.4.1675

[24] Li W, Wang W, Si M, Han L, Gao Q, Luo A, et al The physical state of HPV16 infection and its clinical significance in cancer precursor lesion and cervical carcinoma. Journal of cancer research and clinical oncology. 2008;134(12):1355–61. 10.1007/s00432-008-0413-3 . 18478264 PMC12161717

[25] Gewin L, Galloway DA. E box-dependent activation of telomerase by human papillomavirus type 16 E6 does not require induction of c-myc. Journal of virology. 2001;75(15):7198–201. 10.1128/JVI.75.15.7198-7201.2001 11435602 PMC114450

[26] Chang TC, Yu D, Lee YS, Wentzel EA, Arking DE, West KM, et al Widespread microRNA repression by Myc contributes to tumorigenesis. Nat Genet. 2008;40(1):43–50. 10.1038/ng.2007.30 18066065 PMC2628762

[27] Huibregtse JM, Scheffner M, Howley PM. Localization of the E6-AP regions that direct human papillomavirus E6 binding, association with p53, and ubiquitination of associated proteins. Molecular and cellular biology. 1993;13(8):4918–27. 8393140 10.1128/mcb.13.8.4918-4927.1993 PMC360130

[28] Scheffner M, Huibregtse JM, Vierstra RD, Howley PM. The HPV-16 E6 and E6-AP complex functions as a ubiquitin-protein ligase in the ubiquitination of p53. Cell. 1993;75(3):495–505. . 8221889 10.1016/0092-8674(93)90384-3

[29] Brosh R, Shalgi R, Liran A, Landan G, Korotayev K, Nguyen GH, et al p53-Repressed miRNAs are involved with E2F in a feed-forward loop promoting proliferation. Molecular systems biology. 2008;4:229 10.1038/msb.2008.65 19034270 PMC2600669

[30] Oh ST, Kyo S, Laimins LA. Telomerase activation by human papillomavirus type 16 E6 protein: induction of human telomerase reverse transcriptase expression through Myc and GC-rich Sp1 binding sites. Journal of virology. 2001;75(12):5559–66. 10.1128/JVI.75.12.5559-5566.2001 11356963 PMC114268

[31] Petrocca F, Visone R, Onelli MR, Shah MH, Nicoloso MS, de Martino I, et al E2F1-regulated microRNAs impair TGFbeta-dependent cell-cycle arrest and apoptosis in gastric cancer. Cancer cell. 2008;13(3):272–86. 10.1016/j.ccr.2008.02.013 . 18328430

[32] Nees M, Geoghegan JM, Hyman T, Frank S, Miller L, Woodworth CD. Papillomavirus type 16 oncogenes downregulate expression of interferon-responsive genes and upregulate proliferation-associated and NF-kappaB-responsive genes in cervical keratinocytes. Journal of virology. 2001;75(9):4283–96. 10.1128/JVI.75.9.4283-4296.2001 11287578 PMC114174

[33] Yang CH, Yue J, Fan M, Pfeffer LM. IFN induces miR-21 through a signal transducer and activator of transcription 3-dependent pathway as a suppressive negative feedback on IFN-induced apoptosis. Cancer research. 2010;70(20):8108–16. 10.1158/0008-5472.CAN-10-2579 20813833 PMC3014825

[34] Han J, Huo M, Mu M, Liu J, Zhang J. [miR-497 suppresses proliferation of human cervical carcinoma HeLa cells by targeting cyclin E1]. Xi bao yu fen zi mian yi xue za zhi = Chinese journal of cellular and molecular immunology. 2014;30(6):597–600. . 24909281

[35] Xu J, Wang T, Cao Z, Huang H, Li J, Liu W, et al MiR-497 downregulation contributes to the malignancy of pancreatic cancer and associates with a poor prognosis. Oncotarget. 2014;5(16):6983–93. 10.18632/oncotarget.2184 25149530 PMC4196178

[36] Zhao X, Zhao Z, Xu W, Hou J, Du X. Down-regulation of miR-497 is associated with poor prognosis in renal cancer. International journal of clinical and experimental pathology. 2015;8(1):758–64. 25755771 PMC4348935

