# Supplementary material for: miRNA Expression Profiles of HPV-Infected Patients with Cervical Cancer in the Uyghur Population in China
Source: PLoS One. 2016 Oct 20;11(10):e0164701. doi: 10.1371/journal.pone.0164701 (PMC5072605; doi:10.1371/journal.pone.0164701)
Supplement: S1 Protocol — (DOCX) [file pone.0164701.s001.docx]

**Trial Protocol**

**Patient and tissue samples**

Cervical cancer tissues and uninfected normal mucosa tissues were obtained from the Obstetrics and Gynecology Department of Xinjiang Medical University Cancer Hospital. All tissues were obtained between June 2012 and June 2013 as fresh specimens from surgical resection, and stored at -80°C until use. There were 30 cervical cancer tissues and 26 normal mucosa tissues. All patients were from the Uyghur population of Xinjiang, China. All cervical specimens were confirmed as squamous cell carcinoma through pathological examination. Prior to tissue sampling, patients had not received any chemotherapy treatment. Samples from the control group were determined to be normal by ThinPrep cytologic tests. This study was approved by the Ethics Committee of Xinjiang Medical University Cancer Hospital (K-201343), and informed consent was obtained from all patients.

**Detection of HPV infection**

Total DNA was isolated from 30 cervical cancer tissues using the TIANamp Genomic DNA kit (Tiangen Biotech Co. Ltd, Beijing) according to the manufacturer’s instructions, and DNA concentrations were determined using a NanoDrop 2000 spectrophotometer (Thermo Fisher scientific, USA). PCR was performed on a gradient PCR instrument (Bio-Rad, USA), and the DNA polymerase used was Taq polymerase (Thermo Fisher Scientific, USA). All PCR primers used are listed in Table 1. Detection of HPV infection was first performed by applying primers MY09 and MY11 in the first round of PCR, and the PCR product was then diluted 1:100 before adding primers GP5+ and GP6+ to perform the second round of PCR. For detection of HPV16 subtypes, primers for GP-E6-3F and GP-E6-5R were used in the first round of PCR, and then HPV16Fz and HPV16Rz primers were added to a 1:100 diluted PCR product to perform the second round of PCR. The final product was electrophoresed on 1% agarose gel. For quantitative PCR based on amplified fluorescence of multiple Taqman probes, the pCR-XL-TOPO-HPV16 plasmid was used to construct a standard curve (State Key Laboratory of Biological Resources and Genetic Engineering, School of Life Sciences and Technology, Xinjiang University). Based on the amplified results of the HPV16 plasmid standard, HPV infection of each sample was determined as either an episomal or integrated form.

**MicroRNA microarray analysis**

Six cervical cancer tissues infected with HPV16 were randomly selected along with six normal mucosa tissues. Total RNA was isolated using the Trizol Reagent (Invitrogen, USA) according to the manufacturer’s instructions. RNA concentrations were determined using a NanoDrop 2000 spectrophotometer (Thermo Fisher scientific, USA). RNA quality and integrity were examined using an Agilent 2100 Bioanalyzer (Agilent Technologies, USA). All RNA samples used for miRNA microarrays exhibited a 2100RIN (RNA integrity number) of ≥ 6.0, and ratios of 28S/18S ≥ 0.7. Subsequently, reverse transcription (RT) was performed on these samples using 1.0 mg of total RNA to generate cDNA using the miScript II RT kit (QIAGEN, Germany).

Labeling and hybridization were performed by the Urumqi OE Biotech Company, according to the protocols of the Agilent miRNA microarray system. Scanning of microarrays was completed using the Agilent Scan Control software, and all analyses were performed using the Agilent Feature Extraction software (version 10.7.1.1, Agilent Technologies). Raw data were standardized using the Quantile algorithm, Gene Spring Software (Agilent technologies, US).

**Quantitative real time polymerase chain reaction analysis (qRT-PCR)**

Cervical cancer tissues infected with HPV16 and normal mucosa tissues were randomly selected (n = 20/group), and total RNA was isolated using the Trizol reagent (Invitrogen, USA), according to the manufacturer’s instructions. RNA concentrations were determined using a NanoDrop 2000 spectrophotometer (Thermo Fisher scientific, USA). Total RNA (1 mg) was converted into cDNA using the miScript II RT kit (QIAGEN, Germany). Real-time PCR was performed using SYBR Select Master Mix reagents (Invitrogen, USA) on an ABI Prism 7500 detection system (Invitrogen, USA). The PCR conditions were as follows: denaturation at 95°C for 15 sec, followed by annealing and extension at 60°C for 1 min. The primers for miR-218-5p, miR-17-5p, miR-96, miR-15a-5p, miR-20a-5p, and miR-21-5p were obtained from Tiangen Biotech Co. Ltd (Beijing, China). The other primers are listed in Table 1. The internal control was U6, and miRNA expression level was calculated using the 2-ΔΔCt method.
